# Multi-dimensional impact of COVID-19 on active mobility in urban China: a scoping review of empirical knowledge

**DOI:** 10.3389/fpubh.2024.1398340

**Published:** 2024-05-09

**Authors:** Shengchen Du, Hongze Tan, Hua Gao

**Affiliations:** ^1^Department of Sociology, Tianjin University of Technology, Tianjin, China; ^2^Department of Sociology, Nankai University, Tianjin, China; ^3^Beijing Federation of Trade Unions Cadre College, Beijing, China

**Keywords:** active mobility, physical activity, COVID-19, post-epidemic era, China

## Abstract

Active mobility, such as cycling and walking, is assuming a growing significance in the daily lives of urban residents in China due to its positive impact on health and the environment. The impact of the COVID-19 epidemic has elicited significant changes in behaviors, perceptions, and intellectual viewpoints in this domain, potentially altering residents’ physical activities in the long-term. This scoping review seeks to delve into the multi-dimensional influence of the epidemic on active mobility in urban China. A thorough investigation of English and Chinese studies up to January 2024 was conducted, drawing from articles in Web of Science and the Chinese National Knowledge Infrastructure. Only empirical studies providing knowledge into this subject were selected in the review, which comprised 20 studies in total. This review indicates that the influence of COVID-19 on active urban mobility in China has exhibited contradictory outcomes in terms of behavior. Besides, the experiences during the epidemic have significantly shaped citizens’ attitudes and understanding of active mobility. The repercussions of the epidemic and the ensuing restrictions exacerbate the existing challenges faced by women, particularly those who are married, the older adult, and individuals with low incomes. The results exhibit both resemblances and idiosyncrasies when juxtaposed with prior research conducted in different nations. This analysis also offers valuable insights for improving active mobility across individual, organizational, and socio-political realms. The current state of empirical understanding in this field underscores the need for further research endeavors employing diverse methodological approaches and increased emphasis on the transformations anticipated in the post-epidemic era.

## 1 Introduction

Active mobility, or saying active transportation, is the transport of people or goods, through non-motorized means, based on human physical activity (1), and its best-known forms are walking and cycling. Because of its healthy and environmental benefits, it is an important component of sustainable mobilities (2, 3). In recent years, active mobility has attracted increasing concern all over the world, especially in cities suffering from increasingly serious transportation, health, and environmental issues (4, 5). Urban China is facing an obesity and chronic disease epidemic along with traffic congestion and air pollution due to rapid urbanization, population aging, and unhealthy lifestyles (6). That’s why promoting active mobility is becoming a common governmental response in pursuing sustainable urban development. This is reflected in the implementation of a series of measures represented by urban greenways, pedestrian streets, bicycle-friendly streets, and various kinds of shared bicycles in many cities (7–9).

The outbreak of COVID-19 and its associated constraints have had notable repercussions on the everyday travel patterns of affected citizens (10). This transformation is particularly pronounced within urban areas of China. Leveraging insights gained from the SARS outbreak of 2002–2003, the Chinese authorities have enforced rigorous protocols to limit the movement of their populace in light of COVID-19. Chinese residents witnessed some of the most pronounced and significant alterations in their daily travel routines amidst the epidemic (11–13). A series of empirical studies have discovered the negative relationship between COVID-19, encompassing anti-epidemic policies and measures, and the physical activity levels as well as physical health of Chinese citizens (14–16). Studies in the realm of daily active mobility have revealed that the epidemic has a direct negative impact on the quantity, regularity, distance, and inclination of individuals to engage in active transportation (17–19).

Underlying diminished levels of active mobility, however, some other studies have unveiled a multitude of circumstances. One such instance pertains to the adoption of alternative means of transportation, notably bike sharing, by individuals faced with disruptions in public transit and taxi services during the epidemic. This shift has opened a window of opportunity to encourage more individuals to embrace active travel and to promote greater awareness of the health advantages associated with active transportation (20). Besides, the epidemic has prompted numerous individuals to contemplate the health and environmental advantages of increasing their walking or cycling activities. This alteration in perspective may influence their attitudes toward everyday commuting practices (21, 22). Additionally, the impact of the epidemic on active travel, which entails daily mobility undertaken by diverse individuals for multifaceted reasons, is intricately shaped by sociodemographic variables such as gender, age, income, and other related factors (23, 24). The epidemic might also bring new variations in travel inequalities in affected cities (25, 26). To the best of our understanding, however, a comprehensive evaluation of existing empirical knowledge about how and in what dimensions that COVID-19 impacted Chinese citizens’ daily active mobility is currently lacking in scholarly literature.

The void requiring attention arises from the enduring impact of COVID-19, which represents an extraordinary lived encounter for all individuals impacted over an extended duration. This shared collective experience is poised to exert a profound influence on the resurgence and advancement of urban active mobility in the aftermath of the epidemic (27). Consequently, a comprehensive evaluation of the diverse impacts of COVID-19 on active mobility in Chinese cities is vital for advancing sustainable and healthy urban mobility practices in the aftermath of the health crisis. To address this research need, we conducted a scoping review to explore the existing empirical evidence regarding the multi-dimensional impact of COVID-19 on urban active mobility in China.

## 2 Method

The primary objective of this paper is to examine the empirical knowledge available on the impact of COVID-19 on urban active transportation in China. To this end, we have utilized the scoping review approach as our methodology. A scoping review is considered an apt tool when the studies being reviewed are heterogeneous, making it impossible to conduct a systematic review or meta-analysis on specific research questions (28). Through the scoping review approach, we aim to provide a comprehensive overview of the research scope, results, and gaps in existing studies (29, 30). Specifically, the study examines the following research questions: (1) What behavioral, attitudinal, intellectual, and situational changes have been discovered and analyzed in relation to Chinese urban active transportation? (2) How were these changes discovered and analyzed? (3) How did they emerge and evolve? (4) What insights do current findings provide for our understanding of Chinese active transportation in the post-epidemic era?

### 2.1 Data sources and search strategy

Our inquiry included an extensive review of studies pertaining to the effects of COVID-19 and associated anti-epidemic measures on active transportation in China. Our research scope encompassed empirical studies published in both English and Chinese. On January 29, 2024, we conducted our search for studies published prior to this date.

We conducted a thorough investigation of English language research by utilizing Web of Science (WoS), a comprehensive database that incorporates a variety of indexes such as Science Citation Index Expanded, Social Sciences Citation Index, Arts & Humanities Citation Index, Emerging Sources Citation Index journals and so on. It includes all the concerned disciplines of this review, like public health, transportation, urban studies, environmental science and so on. Our search was constrained to peer-reviewed empirical studies that were relevant to our topic. To limit our search further, we used an array of keywords, including COVID-19 and terms related to active transportation, such as active mobilities, active transportation, active travel, cycling, bike, bicycle, walking, pedestrian, and location (China). We specifically focused on walking and cycling, as these two modes are basic constitutions of active transportation in current China. To ensure an exhaustive search, we did not restrict the categories of studied participants.

**Figure 1:**
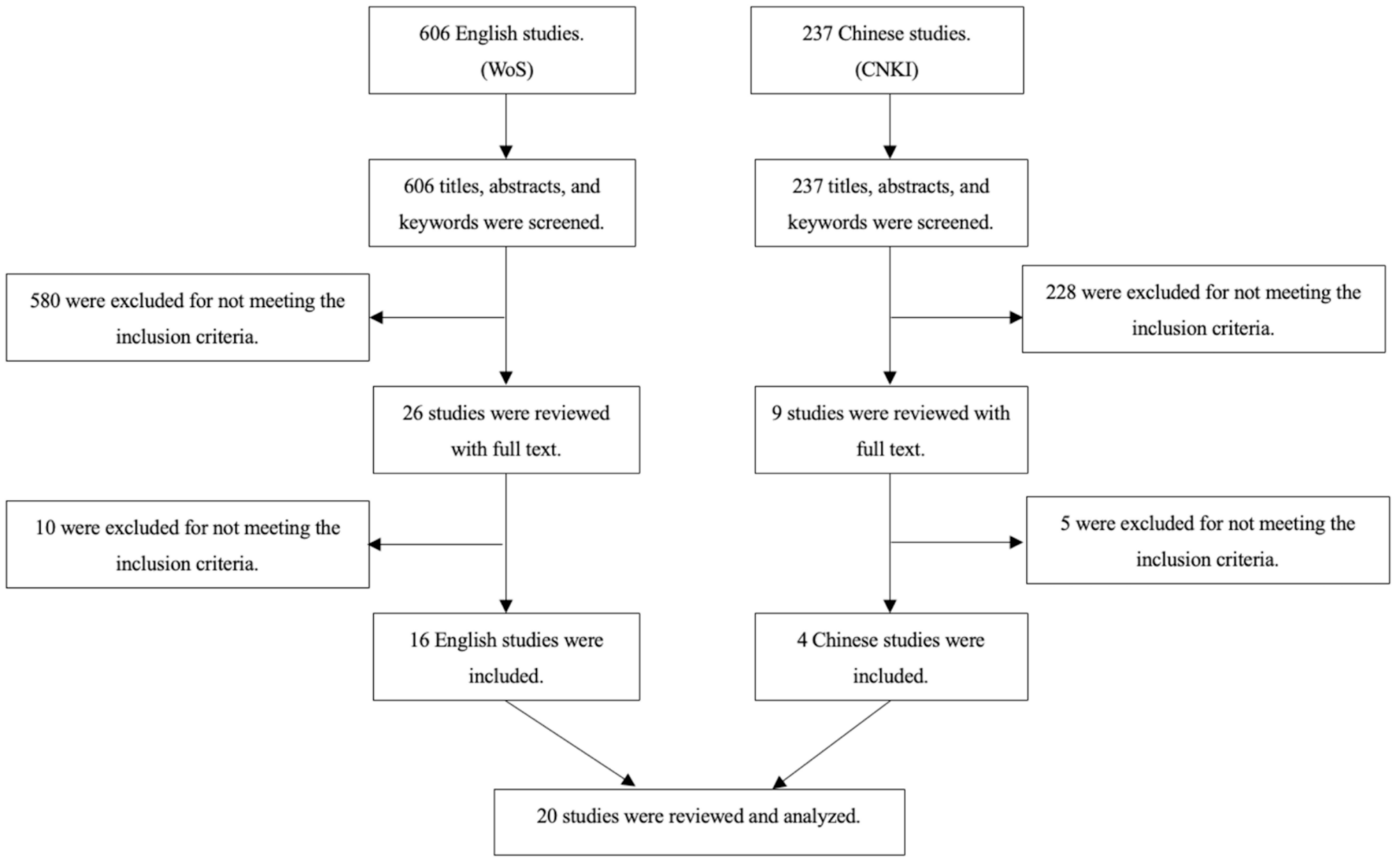
Search and review procedures.

Our investigation consisted of conducting a thorough exploration within the Chinese National Knowledge Infrastructure (CNKI) database for empirical studies written in the Chinese language. CNKI is the most widely used and the most authoritative academic database in China (31). Our initial search of relevant keywords translated into Chinese yielded much fewer studies than those available in the English language. Consequently, we adopted a more inclusive approach to our Chinese retrieval to enhance our search results. We incorporated all relevant studies from a search of COVID-19 (“*Yiqing*” / “*Xinguan*”) accompanied by bike/bicycle/cycling (“*Zixingche*”), or walking/pedestrians (“*Buxing*”). Since most of the articles in CNKI were location-specific to China, we refrained from utilizing any location-based keywords. The search and review procedures show in Figure 1.

### 2.2 Inclusion and exclusion criteria

This review incorporated empirical studies examining active mobilities in China following the emergence of COVID-19. These modifications included changes in Chinese residents’ travel behavior, perceptions, and awareness, as well as modifications in transport-linked infrastructures and built environments, both movable and immovable. Qualitative, quantitative, or hybrid empirical data collection and analysis methods were employed in these studies, with study subjects consisting of Chinese residents with some sort of visible or hidden travel alterations. Given the considerable economic and socio-political disparities between mainland China and Taiwan, Hong Kong, and Macao, this review excluded research carried out in these areas. Master’s theses, doctoral dissertations, and conference papers were also omitted from the review because most of them were not peer-reviewed, and it was challenging to assess their research quality.

### 2.3 Review process

Initially, the authors conducted a thorough review of the titles, keywords, and abstracts of the articles that were selected. Only studies that met the criteria for inclusion received further screening. The authors then undertook a full-text review of the selected studies to establish the final list of included and excluded studies, a process that was conducted independently by two of the authors. If any disagreement between the authors arose, a discussion ensued until a consensus was reached. A detailed depiction of the review procedures was provided in Figure 1. The search yielded a total of 606 research articles in English, of which 580 studies were excluded based on the inclusion criteria after the title, abstract, and keywords screening. A further 10 studies were rejected after the full-text review as they did not meet the required eligibility criteria. Therefore, 16 studies in English qualified for inclusion after the full-text review. Additionally, a total of 237 articles in Chinese were initially identified, of which 228 studies were excluded after the title, abstract, and keywords screening. After a full-text review, four studies in Chinese met the eligibility criteria for inclusion while five studies were excluded. Thus, a total of 20 studies (16 in English and four in Chinese) were included in this scoping review.

We conducted a coding analysis on the selected articles. Studied travel mode (cycling and/or walking), research subject population, study location, research method, covered pandemic period (for the case city/cities), socioeconomic indicators, and active transportation-relevant behavior, attitude, knowledge, and material/facilities changes were coded. Three authors conducted independent coding, with any discrepancies being thoroughly discussed and resolved through consensus after consultation.

### 2.4 Limitation of method

The research relies on data sources from SSCI and CNKI. While prior studies have validated the relative representativeness and reliability of these databases for conducting systematic reviews in each language (31, 32), it is acknowledged that they do not encompass all studies pertinent to the field of active mobility. Consequently, this research overlooks studies published in journals not indexed by these databases. Furthermore, the study’s emphasis on credibility is restricted to published peer-reviewed research, excluding other forms such as academic dissertations, working papers, and research reports. These methodological constraints represent areas for improvement in future research endeavors.

## 3 Results

### 3.1 Characteristics of existing research

Of all the 20 studies, six studies focused on walking only (18, 21, 33–36), eight focused on cycling only (17, 22, 24, 37–41), and another six studies included both cycling and walking (23, 42–46). Bike-sharing is a concerned research topic that five studies focused only on it (17, 22, 24, 37, 41), three of which were about dock-less bike-sharing (DBS) (17, 22, 37), one about station-based bike-sharing (SBBS) (24), and one about both kinds (41). 13 studies focused on general urban residents (17, 22–24, 37–45), while another of seven studied specific research populations, like youths (33, 35, 36, 46), middle-aged and older adult people (18, 21) and disabled people (34). The earliest study was published in October 2020.

A survey by questionnaire was the most used method as 13 studies adopted this method (18, 22, 23, 33–36, 38, 40, 43–46), with the sample size range from 127 (22) to 10,082 (35). Secondary analysis of existing data is another commonly used research method based on data collected from relevant companies (*n* = 4) (17, 24, 37, 41). There was one study that collected and analyzed citizens’ daily steps via a smartphone application (18). Only one research study utilized a qualitative research approach through the execution of in-person interviews (21).

**Figure 2:**
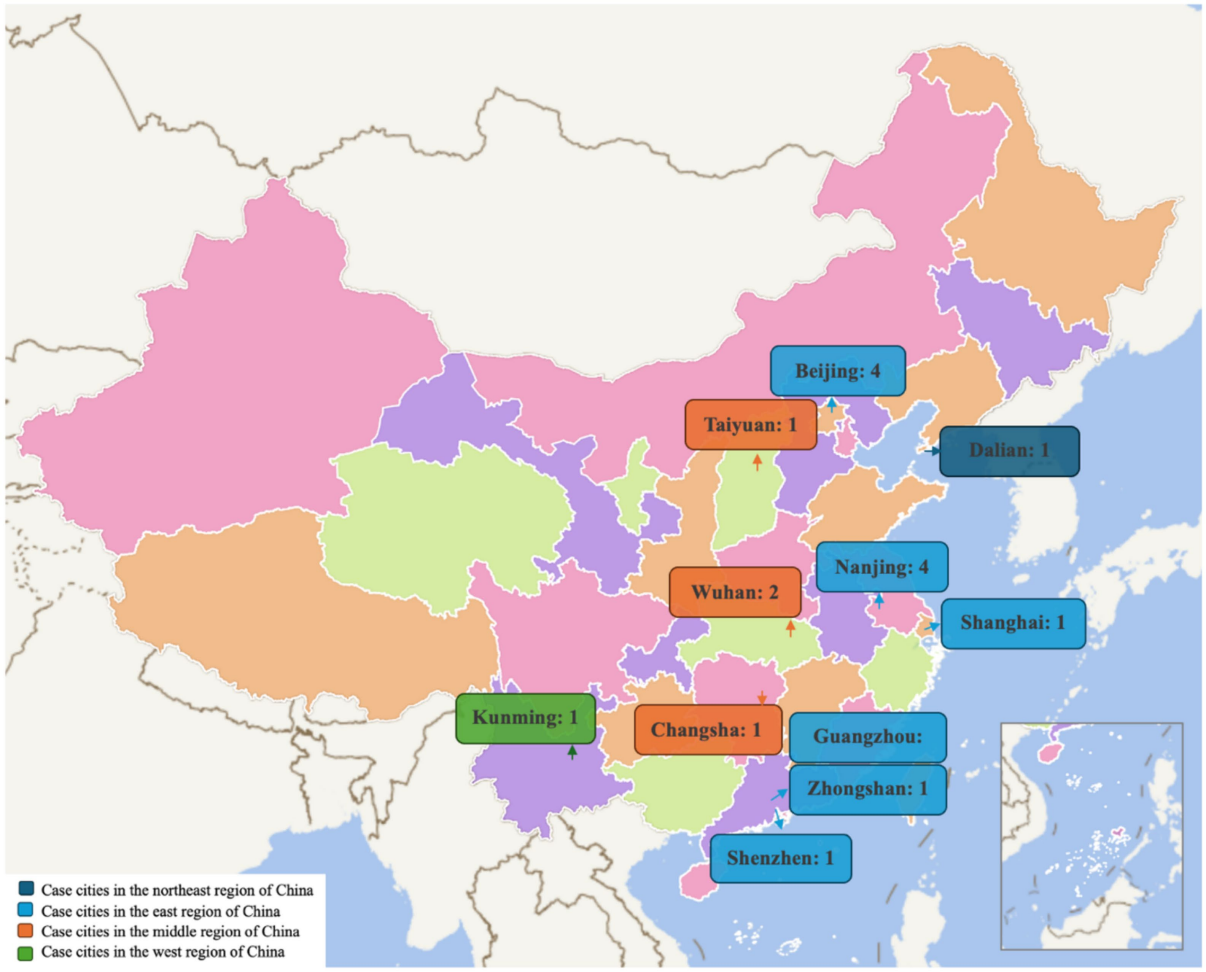
Locations of the studied case cities. This figure is made by the authors based on the open-source map provided by National Database for Geographical Names of China. The division of four regions (west, east, middle, northeast) was based on the classification standard promulgated by the government (https://www.stats.gov.cn/zt_18555/zthd/sjtjr/dejtjkfr/tjkp/202302/t20230216_1909741.htm, accessed 2 April 2024).

Regarding the location of the studies, of the 20 studies, three were national surveys that did not focus on a certain case city (23, 35, 46), while the other 17 were conducted each in a single city (Figure 2). There were 11 studies that were conducted in cities in the eastern region with well-developed economies, such as Beijing (*n* = 4) (36, 38, 43, 45), Shanghai (*n* = 1) (37), Nanjing (*n* = 3) (22, 24, 41), Zhongshan (*n* = 1) (44), Guangzhou (*n* = 1) (39) and Shenzhen (*n* = 1) (40). Wuhan, the city in the middle region that endured the initial outbreak of the epidemic in China, attracted two studies’ attention (17, 42). Changsha and Taiyuan are another two middle region cities that each got one study’s attention (18, 34). There was one study conducted in Kunming, a city located in the western region (21). Another one study was implemented in Dalian (33), which was in the northeast region.

In terms of covered epidemic phases of the empirical studies, due to different cities experiencing different periods and durations of the epidemic, we did not adopt a unified time division. Relatively, we divide the epidemic experience of each into three basic phases: pre-pandemic (before the outbreak of COVID-19, generally before the end of 2019), outbreak phase (the initial and most severe stage of the epidemic in the case city, which was also the most strict stage of epidemic control; for national studies, from January 20th to March 10th, 2020 as defined by State Council of China), and the recovery phase (the “new normal” period after the outbreak). In fact, there should be a post-epidemic era that regards the period after the actual cancelation of epidemic control in China on December 7th, 2022, marked by the release of the “New Ten Rules” of COVID-19. But as all current studies were empirically conducted before this phase, we mainly divided all studies into the above three categories. As changes caused by the epidemic were the key concern, 17 out of 20 studies covered more than one period to conduct certain comparisons. Of the 17 studies, seven covered both pre-epidemic and outbreak phases (18, 22–24, 36, 38, 41), three covered outbreak and recovery phases (42, 44, 45), two compared pre-epidemic and recovery phases (34, 37), and five covered all pre-epidemic, outbreak, and recovery phases (17, 35, 39, 40, 46).

Of the 20 studies, 16 involved findings and explorations on unequal active mobilities among different populations in the context of the epidemic. The two most concerned demographic factors were gender (*n* = 13) (18, 21–24, 33, 35, 36, 40, 41, 44–46) and age (*n* = 9) (18, 21–24, 40, 41, 44, 45), followed by income (*n* = 3) (23, 40, 42), and one study focused on visually impaired people (34). Regarding the language, four were published in Chinese. The other 16 were in English. The characteristics of the reviewed empirical studies were shown in Table 1.

**Table 1:** Characteristics of empirical studies about the impact of COVID-19 on urban active transportation in China.

Author	Analyzed active mobility mode	Studied group of population	Data collection method	Location	Covered epidemic phases (in the studied city/area)	Inequality dimension	Language
Wang et al. (2022)	Walking and cycling	General urban residents	A survey by questionnaire (*N* = 9,010)	Wuhuan, Hubei (Middle)	Outbreak and recovery phases	Income	English
Li and Xu (2022)	Cycling (DBS)	General urban residents	The dataset was provided by Meituan Bike Company	Wuhuan, Hubei (Middle)	Pre-epidemic, outbreak, and recovery phases	Residential area	English
Chen et al. (2022)	Cycling (SBBS)	General urban residents	SBBS smart card data in Nanjing, China provided by Nanjing Public Bike Company	Nanjing, Jiangsu (East)	Pre-epidemic and outbreak phases	Gender and age	English
Barbieri et al. (2021)	Walking and cycling	General urban residents	Online survey (*N* = 9,394) in 10 countries, including China	China (a national study)	Pre-epidemic and outbreak phases	Gender, age, and income	English
Hua et al. (2021)	Cycling (both kinds)	General urban residents	(1) SBBS trip data were provided by Nanjing Public Bike Company; (2) DBS trip data were provided by Nanjing Transportation Bureau	Nanjing, Jiangsu (East)	Pre-epidemic and outbreak phases	Gender and age	English
Zhou et al. (2022)	Cycling (DBS)	General urban residents	The riding records were provided by Meituan Bike Company	Shanghai (East)	Pre-epidemic and recovery phases	\	English
Liu et al. (2022)	Walking	University students	A survey by questionnaire (*N* = 429)	Dalian, Liaoning (NorthEast)	Outbreak phase	Gender	English
Zhang et al. (2022)	Cycling (including bike-sharing)	General urban residents	A survey by questionnaire (*N* = 1,007)	Beijing (Esat)	Pre-epidemic and outbreak phases	\	English
Wang et al. (2020)	Walking	Middle-aged and older people (age ≥ 40)	Daily steps were collected via a smartphone linked to WeChat (*N* = 3,544)	Changsha, Hunan (Middle)	Pre-epidemic and outbreak phases	Gender, age, and education	English
Yu et al. (2022)	Walking	University students	A survey by questionnaire (*N* = 1,460)	Beijing (Esat)	Pre-epidemic and outbreak phases	Gender	English
Yang et al. (2023)	Walking	Youths (aged 19.8 ± 2.3 years)	A survey by questionnaire (*N* = 10,082)	China (a national study)	Pre-epidemic, outbreak, and recovery phases	Gender	English
Liu et al. (2021)	Walking	Older adult people (older than 65)	A qualitative exploration (186 families with a total of 248 older adult people)	Kunming, Yunnan (West)	Outbreak phase	Gender and age	English
Shu et al. (2022)	Walking and cycling	General urban residents	A survey by questionnaire (*N* was not introduced) + field observation	Beijing (Esat)	Recovery phase	\	English
Zhang et al. (2024)	Walking	Visually impaired people	A survey by questionnaire (*N* = 218)	Taiyuan, Shanxi (Middle)	Pre-epidemic and recovery phases	Disabled people	English
Zhou et al. (2021)	Walking & cycling	Youths (at post-mandatory education level)	A survey by questionnaire (*N* = 8,115)	China (a national study)	Pre-epidemic, outbreak, and recovery phases	gender	English
Xie et al. (2023)	Cycling (DBS)	DBS users in Nanjing	A survey by questionnaire (*N* = 127)	Nanjing, Jiangsu (East)	Pre-epidemic and outbreak phases	Gender, age, and occupation	English
Xiang et al. (2023)	Cycling, (including bike-sharing)	Adult commuters	A survey by questionnaire (*N* = 600)	Shenzhen, Guangdong (East)	Pre-epidemic, outbreak, and recovery phases	Gender, age, and income	English
Hu et al. (2021)	Walking and cycling	General urban residents	A survey by questionnaire (*N* = 318)	Zhongshan, Guangdong (East)	Outbreak and recovery phases	Gender, age, and occupation	Chinese
Wei and Liu (2022)	Walking and cycling	General urban residents	A survey by questionnaire (*N* = 1,290)	Beijing (Esat)	Outbreak and recovery phases	Gender and age	Chinese
Ma (2022)	Cycling	General urban residents	Secondary analysis of existing statistical data from previous studies	Guangzhou, Guangdong (East)	Pre-epidemic, outbreak, and recovery phases	\	Chinese

### 3.2 Studies on behavioral changes in active mobility

The scholarly investigation revealed distinct behavioral shifts in active transportation within China across various phases of the epidemic, as evidenced by all 20 studies. These scholarly inquiries unveiled a paradoxical phenomenon in the realm of Chinese urban active mobility induced by the COVID-19 outbreak. While a reduction in the overall volume, distance, and frequency of active travel among citizens was commonly observed due to apprehensions surrounding the epidemic and implementation of lockdown measures, there was a simultaneous elevation in the utilization and prominence of active mobility during this crisis period.

Of all the 20 studies eight analyzed the changes in walking behaviors in the outbreak period. At the national level, citizens saying they never walked during the epidemic increased by 15.5% (23). Daily steps of Changsha residents aged≥40 years dropped significantly, as their mean daily steps dropped from 8,097 to 5,440 and the prevalence of low daily steps increased from 3 to 18.5% (18). In Zhongshan, residents mainly travel short distances during the outbreak, with walking being the main mode of transportation (44). For the youths, significant decreases were also generally observed in their daily walking behaviors (35, 46). For university students in Beijing, the epidemic produced a 24.36% reduction in total weekly minutes of walking (36). Nevertheless, for university students in Dalian, no correlation was found between the impact of the COVID-19 pandemic and walking behavior (33), which is contrary to the finding in Beijing.

Regarding cycling behavioral change in the outbreak period, seven studies brought us interesting findings under the travel decline preface. On the one hand, it is obvious that cycling decrease significantly because of the epidemic. For instance, in Nanjing, the number of station-based bike-sharing (SBBS) users have been hit hard by the outbreak (22, 24), and SBBS trips in this city fell by 72% (41). SBBS trips at all stations declined, and most stations experienced a drop of around 70%. The trips of another kind of sharing bicycle – dock-less bike-sharing (DBS) – in this city even fell by 82% (41). In Wuhan, a significant decrease in bike-sharing trips also emerged during the outbreak (17).

On the other hand, studies also pointed out the positive functions of cycling and its relative increase in the proportion of daily travel during the epidemic. During the epidemic, especially in the outbreak phase, cycling played a key role in replacing bus and subway (40). Before the epidemic, the complementary role of cycling was more important than its substitutive role, but during the epidemic, the substitution role of cycling for public transit was enhanced. For instance, the daily trips of Nanjing Metro fell by 95%, which is much more than the drop in bike-sharing trips in this city. The average travel distance of SBBS increased by 32%, and the average travel distance of DBS increased by 16% (41). Furthermore, while the overall travel duration and travel distance of DBS users decreased after the epidemic, the trip frequency of them increased as the travel duration increased (22). And no evident decline in the proportion of commuters in this city was found (from 36.6 to 34.8%) (24). In Beijing, the proportion of bike-sharing increased by 0.89% (38). The user base of DBS in Shanghai increased during the epidemic (37).

The function of cycling also changed during the epidemic. Before the outbreak, the main function of cycling is commuting. It could be proved by obvious morning and evening cycling peaks between 7 am–9 am and 5 pm–7 pm in cities before the epidemic in Wuhan, which, however, were replaced by a unimodal structure with cycling activities concentrated between 11 am and 2 pm during the outbreak phase (17). This change indicated that the purposes of cycling were more diverse, and cycling played a more important role in residents’ daily life and travel. This can also be supported by the relatively low decrease in independent cycling trips compared with cycling and public transit cooperation trips in Nanjing, which reflected that the major role of cycling changed from cooperating with public transport for commuting to independently providing more categories of mobility services (41). Cycling was more adopted for shopping, scenery, health care, and other travel demands (17, 24, 41).

Regarding active transportation changes in the recovery phase, two studies explored walking changes in this stage: one study pointed out that the proportion of walking in all travel within a district in Beijing was 19% (43); another one study pointed out that for the visually impaired people in Taiyuan, despite the absolute amount of walking decreased, the proportion of walking in their daily travel increased from 35.6% (pre-epidemic) to 42.8% (during the epidemic). There were five studies analyzing cycling changes in this period. In general, due to the advantage of cycling in allowing for social distance and being more useful for relatively long-distance travel, the recovery of it is significant and quick. For instance, in Wuhan, the recovery rate of passenger volume reached 39.80% by June 2020, while the recovery rate of sharing bicycles came to 104.31%, which returned to the level before the epidemic. And by October 2020, the recovery rate of total passenger volume reached 71.40%, while the recovery rate of sharing bicycles became 260.64% year on year (17). In Shanghai, by June 2020, the number of rush-hours sharing bicycle users increased by 2%, and the number of rush-hour rides increased by 4%, compared with those in October 2019 (37). In Guangzhou, the share of trips by owned bicycles in all urban daily travels in 2021 is 11.7%, higher than that (9.18%) in 2019 before the outbreak, and for sharing bicycles, the change is from 3.43% in 2019 to 6.01% in 2021 (39). The substantiation function of cycling for relatively short distance travel was also experienced by many citizens in Shenzhen (40), which may bring in more cycling in the post-epidemic era. Therefore, it seems that urban cycling might enjoy an increase in the recovery phase and even in the post-epidemic era. Nevertheless, this prediction may not be applicable to SBBS. As in Nanjing, the monthly usage of SBBS continued to decline and did not return to pre-epidemic volumes, and scholars inferred that people may become less willing to use shared modes of transportation in the post-pandemic era (24). The brief summary of behavioral changes in active mobility during the epidemic shows in Table 2.

**Table 2:** Summary of behavioral changes in active mobility during the epidemic.

Location	Active mobility amount (the number of trips, distance, and frequency, compared with the pre-epidemic phase)		Active mobility function (the purpose of active travel)	Reference
	Outbreak Phase (compared with the pre-epidemic phase)	Recovery Phase (compared with the outbreak phase)		
Wuhuan	Decrease	Increase	More diverse	(17, 42)
Nanjing	Decrease (But the proportion of bike-sharing increased.)	Decrease (SBBS)	More diverse	(22, 24, 41)
Shanghai	Decline	Increase	\	(37)
Dalian	No change	\	\	(33)
Beijing	Decrease	Increase	\	(8, 33, 36, 43, 45)
Changsha	Decrease	\	\	(18)
Kunming	Decrease	\	\	(21)
Taiyuan	\	Increase	\	(8)
Shenzhen	\	\	More diverse	(40)
Zhongshan	Decrease	Increase	More diverse	(44)
Guangzhou	\	Increase (compared with the pre-epidemic phase)	More diverse	(39)
National level	Decrease	\	\	(3, 23, 35)

### 3.3 Studies on changes in attitudinal and knowledge-based dimensions of active mobility

Exploring the shifts in public perception and understanding of active transportation in light of the COVID-19 epidemic can yield valuable insights into the latent and enduring impacts of the outbreak. Among the 20 studies reviewed, half specifically examined this aspect. While an inherent negative correlation exists between pandemics and travel patterns, it is noteworthy that enhanced awareness of the epidemic tended to foster a more favorable attitude toward walking and cycling among residents.

As COVID-19 is mainly transmitted through the air, most citizens believed that keeping a safe distance from others is an effective means to avoid infection (22), which made active travel modes more favored during the epidemic period. At the national level, walking and cycling were perceived as low probability of contracting COVID-19 by Chinese citizens in the outbreak and recovery phases (23). In Wuhan, after the outbreak, citizens tended to make various preparations for non-motorized travel, and with the upgrading of epidemic control measures, citizens’ walking and riding maximum willingness distances were both continuously increasing (42). In Shanghai, after experiencing the outbreak of the epidemic, the enhanced user base improves people’s confidence about the long survival of the bike-sharing industry, and citizens showed increasing positive value of bike-sharing as a complement to public transit in the upcoming post-epidemic era (37). But the epidemic also brought certain negative experience in the active travel of some groups of people, which affected their attitude regarding active travel. For instance, for visually impaired people in Tainyuan, the unwillingness to travel using blind track increased from 26% (pre-epidemic) to 41.8% (during the epidemic). This may partially because that visually impaired people need assistance or support from others while walking crossing the road, and the lockdown during COVID-19 caused their dissatisfaction (34).

It is significant that the relationship between attitude, knowledge, and behavior regarding active travel was moderated by the severity of the epidemic. For instance, during the outbreak phase in Beijing, the more citizens understood epidemic prevention policies, the greater the likelihood of choosing to walk, but this kind of positive relation appeared to weaken in the recovery phase (45). Similarly, for the university students in Dalian, although there was a generally positive attitude among them, the impact of the epidemic was negatively correlated with the walking attitude, which had a significant impact on students’ walking behavior on weekends (33). In Beijing, among all kinds of sharing mobilities, bike sharing was perceived as the second safe but the second to last comfort by citizens, and when the perceived epidemic severity was relatively low, citizens’ preference for cycling significantly increased (38).

For some citizens, especially the older adult, the role of active mobility as an activity by itself was more important during the epidemic. After the outbreak, because all public spaces were closed, walking almost offered the only opportunity for many older citizens to interact with non-family members (21). Active mobility served as an important, if not the only, part of social life for many older adult citizens, so it’s understandable that they show resistance to travel restrictions.

### 3.4 Inequalities in active mobility during the epidemic

**Figure 3:**
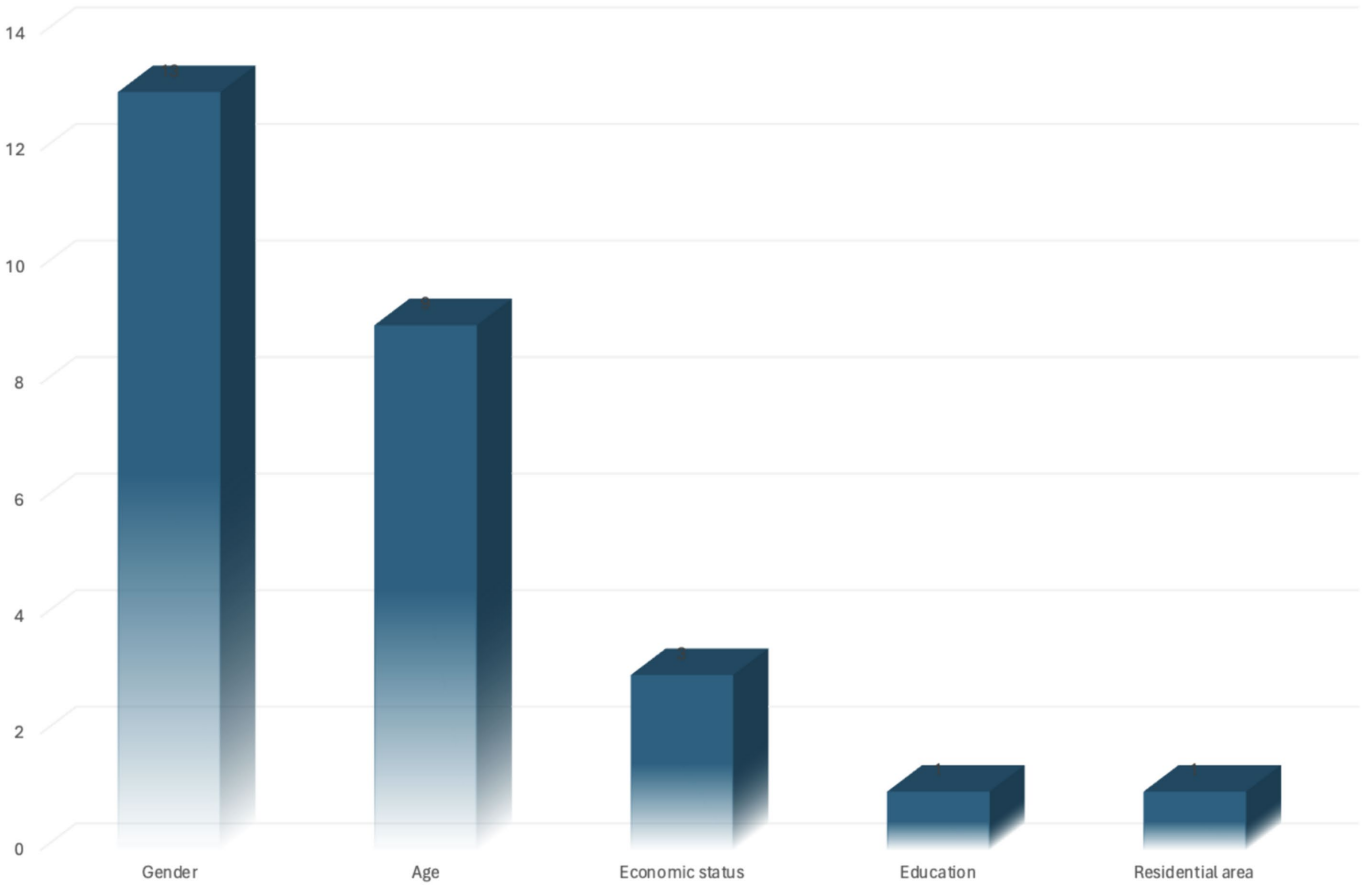
Analyzed inequality dimensions in active mobility in urban China during the epidemic.

A series of studies explored transportation-related social equity among different demographic groups due to the COVID-19 epidemic (Figure 3). Gender is the most concerned dimension of existing studies in exploring the inequalities in active transportation during the epidemic. Out of 20 studies, 13 found changes in this dimension. In general, the outbreak accelerated more decline in the daily active travel of females compared with males, which was reflected in various behavioral changes. In Nanjing, before the outbreak, the SBBS trips of females and males are roughly equal, but the female trip proportion fell from 47 to 43% after the outbreak (41), and there was also a significant decline in the proportion of female SBBS commuters (24). In Changsha, females were associated with a higher prevalence of frequent low daily steps, which were more pronounced during the epidemic period (18). In both Beijing and Zhongshan, after the outbreak, males were more willing to conduct cycling compared with females (44, 45).

The reason that females were more likely to be affected by the epidemic might not be because they were more afraid of the epidemic, as gender had no significant impact on their attitudes regarding active travel (33). It might be because during the epidemic more females had to stay at home to care for the children who studied at home as schools were closed (41). This was partially proved from the reverse side as that for those young and unmarried females, like university students, the epidemic caused less decrease in physical activity compared with males (36), although the young females even conducted more moderate−/vigorous-intensity housework during the epidemic (35). Besides, as females generally had relatively low participation in cycling and used it more as the complement of public transport (22), the huge decline in public transportation travel during the epidemic may also contribute to the low active mobility of females.

Age is another important dimension regarding the active travel inequality issue, as nine out of 20 studies explored this dimension. The relationship between age and daily active travel is a little complex. For the youths, as schools were all closed during the epidemic, it is understandable that their daily travel dropped significantly and heavily (24, 41). For the older adult, however, the impact of the epidemic was multi-dimensional. On the one hand, for the health concern, the absolute daily active travel of the older adult decreased, which is consistent with the overall situation of the whole society. For instance, in Changsha, the daily steps of Changsha elder residents dropped significantly during the outbreak (18). Nevertheless, some studies discovered that the aged depended more on active mobility and conducted relatively more active travel compared with the young citizens. For instance, during the outbreak in Nanjing, the proportion of SBBS trips of older adult users increased, and their trip amount was least affected by the pandemic (41). Similarly, in Zhongshan, older citizens were more likely to choose walking for travel after the outbreak (44). In Kunming, most of the older citizens routinely performed outdoor physical activities in the first month of the outbreak, whilst only much fewer young people went out regularly during that time (21). However, there are other studies with different findings. For instance, for the users of DBS in Nanjing, the variable of age had a significantly negative effect on cycling after the epidemic, as the older adult were more sensitive to the possibility of infection risk (22).

This phenomenon reflected the dual dilemma of the older adult in modern digital society as a partial result of the digital divide (47). First, as the older adult were generally less technology-savvy and were less engaged in Cyber-society, the fill of their various material and emotional needs relied more on offline face-to-face interactions. So, during the epidemic, unlike the young people who could conduct shop, work, socialize, and entertainment at home, the older adult had to go out. Especially those elder people who did not live with their children. Second, the epidemic greatly promoted the “digital reform” of the Chinese urban transportation system, and a smartphone is a necessary device for using various transportation modes, especially public transit (21). Therefore, for most elder residents, it was very inconvenient and even unfeasible to use other travel modes besides walking and cycling.

There were three studies exploring the differences in active travel between groups of different economic statuses. There was a significant positive correlation between monthly income and the choice of active mobility during the epidemic. For instance, a study in Beijing found that after the outbreak, the higher the monthly income of travelers, the stronger their willingness to conduct cycling for daily travel (45). In Zhongshan, during the outbreak period, citizens with higher economic status showed more willingness to choose walking rather than electric bicycles (44). Similarly, in Shenzhen, those citizens who tend to ride bicycles for commuting during the epidemic have less income than average (40). It might be because the upper-middle income groups could work from home and enjoy higher economic resilience to stop working, so they mainly conducted short daily travel during the epidemic (42). This is partly proved by the fact that coming into the recovery phase, the relationship between economic conditions and the conduct of active mobility turned negative (44).

Besides the above three dimensions, other dimensions like education and residential area were also concerned by some studies. For instance, a research investigation revealed that individuals possessing a university degree or higher exhibit a reduced incidence of consistently low daily step counts in the period preceding an epidemic, in contrast to those with a high school education or less. However, this disparity was not observed during the epidemic phase (18). Cycling in the urban area of Nanjing was less impacted by COVID-19, and the suburban area was relatively more impacted (41). In the recovery phase in Wuhan, however, more cycling neighborhoods emerged in suburban areas, and the percentage of ridership in denser areas tends to decrease, which showed a trend toward decentralization and localization (33).

## 4 Discussion and applications to further research

The onset and persistence of the COVID-19 outbreak have had a profound impact on the daily routines and engagements of Chinese citizens. Among the various aspects affected, physical activity stands out as one of the most significantly influenced areas (36, 46). Research has revealed a marked reduction in physical activity levels among Chinese individuals due to the outbreak, leading to adverse effects on their overall well-being (12, 14). Unlike indoor exercises focused on health benefits (16), active mobility involves outdoor movements for diverse purposes and has been particularly impacted by the outbreak compared to other forms of physical activity. A comprehensive examination of the shifts in Chinese citizens’ active mobility patterns during the outbreak not only enables a deeper understanding of the societal repercussions of the crisis but also offers valuable insights on promoting sustainable active mobility practices in the post-epidemic era. Based on this concern, this systematic scoping review addresses the impact of COVID-19 on active mobility in China through an empirical lens. Following rigorous screening procedures, 20 empirical studies were meticulously chosen from a pool of 606 English articles and 237 Chinese articles. A comprehensive analysis of these studies reveals significant insights into the subject matter.

First, the impact of COVID-19 on Chinese urban activity exhibited paradoxical effects on the behavioral dimension, which is consistent with scholars’ analysis of the complexity of the epidemic impact (19, 27, 48). While the epidemic led to a decrease in active mobility in urban areas, with reduced frequencies and distances covered in walking and cycling trips, there was also an observed increase in the adoption of active mobility practices among Chinese citizens. The proportion of active mobility in daily travel rose during the outbreak phase, with individuals engaging in active travel for a variety of purposes more frequently than before the epidemic. Bike-sharing, as a form of active mobility in the digital age, emerged as a significant mode of urban transportation during the epidemic, with an increase in the proportion of bike-sharing trips, distance traveled per trip, and diversity of trip purposes. The recovery rate of bike-sharing trips surpassed that of other transportation modes, hinting at a potential rise in the acceptance and use of bike-sharing among Chinese citizens in the post-epidemic era.

Second, the experience amid the epidemic significantly influenced Chinese citizens’ attitudes and understanding of active mobility. Throughout this period, citizens in China not only acknowledged the practical significance (49, 50) of active mobility, but also recognized it as a form of physical and social engagement. Active travel transcended its traditional role of transporting individuals and goods from one point to another, providing travellers with physical, social, and emotional benefits, particularly benefiting certain demographics like the older adult. This suggests that the epidemic presented an opportunity to transition toward the “new mobilities paradigm” and foster a more supportive environment for active mobility development. However, it is important to note that negative connotations toward mobility emerged during the epidemic, particularly regarding “non-essential travel” (a term commonly used in China by the government during the epidemic). The evolving attitudes and knowledge pertaining to active mobility among Chinese citizens may have enduring and profound implications, surpassing short-term behavioral changes.

Third, the influences of the epidemic and associated restrictions tend to compound and exacerbate the existing disadvantages faced by (married) women, the older adult, and low-income individuals in daily active transportation. Women, especially the married ones may experience reduced mobility due to the uneven distribution of childcare duties within couples. The older adult, on the other hand, encounter challenges related to the digital divide and transportation disparities, leading to increased active travel during the epidemic phase but inconvenience and exclusion during the recovery period. Low-income groups, with limited economic resilience, are compelled to undertake medium to long-distance travels even amidst the outbreak, resulting in comparatively lower levels of active transportation compared to their higher-income counterparts. Therefore, a nuanced understanding of active travel patterns during the pandemic and beyond is crucial, rather than simply advocating for increased active mobility as a solution. It is essential to recognize that the promotion of active travel does not automatically translate to enhanced travel equality across different socioeconomic groups.

The extant literature on active mobility in urban China exhibits both similarities and distinctions when compared to related studies in other nations. Notably, the research conducted in urban China has confirmed the simultaneous effects of COVID-19 on active mobility observed in other countries, like in the US (51–53), Iran (54), Greece (55), Australia (56), Bangladesh (57), Serbia (58), South Korea (59), and so on. This is evidenced by a reduction in the total amount of active travel, coupled with an increase in the proportion of active travel utilized in citizens’ daily travel patterns. The observation underscores a second commonality, namely the emergence of a potential window of opportunity to advocate for increased active mobility due to the epidemic as discovered by studies in Europe (60, 61), the US (62) and Latin America (63, 64). This situation may prompt citizens to acknowledge and prioritize the health and environmental advantages associated with active mobility. Furthermore, research conducted in urban China validates the altered landscape of active mobility equality resulting from the epidemic, particularly affecting vulnerable demographics such as females, the older adult, and individuals with lower incomes (56, 64–66).

The review also identifies unique characteristics in urban China that have not been observed in other nations. First, due to enduring stringent measures over an extended period, Chinese residents have demonstrated a notably more substantial and favorable shift in their perception toward active mobility. This is evidenced by the swift resurgence in active mobility levels and the wider range of purposes for active transportation during the epidemic, a trend distinct from the developments seen in South Korea (59), Hungary (67), Canada (66) and Latin America countries (63). Second, the significance of cycling, particularly the utilization of shared bicycles like DBS, stands out in China amidst the epidemic. Unlike the limited impact observed on SBBS as noted in studies across different nations (24, 67), the epidemic has had a predominantly positive and enduring effect on DBS (17, 41). This underscores the considerable promise of such forms of active mobility in the global landscape post-epidemic. Third, the impact of epidemics on gender dynamics in active mobility in China underscores the precarious position of married women with children. This is different with the reduction in the gender gap discovered in France (65) and Canada (66). It may reveal the disparities in the allocation of family duties within dual-income households in contemporary urban China, necessitating further empirical investigations. Finally, studies conducted in other countries have underscored that the significance of the enduring favorable impact for active mobility provided by the epidemic-induced opportunity window can yield, contingent upon the provision of suitable preconditions (60, 61, 67). This underscores the necessity for the implementation of policies, regulations, and infrastructures that promote active mobility even amidst the epidemic (62, 64). Nevertheless, there has been a notable dearth of such initiatives in urban China, even during the epidemic recovery phase. Consequently, it is imperative to undertake additional active mobility initiatives across various levels in the contemporary post-epidemic period to capitalize on the opportunity window before it eludes.

This analysis enriches our understanding of the collective experience of Chinese residents throughout the epidemic period and presents valuable perspectives for advancing active mobility in the post-epidemic era. On an individual level, the first-hand encounters during the epidemic serve as tangible assets for advocating and sustaining increased mobility in the future. Many individuals are likely to consider walking and cycling as viable and health-conscious options for their daily commuting needs. Additionally, the increased integration of smart technology with active mobility can be attributed in part to the impact of COVID-19. The utilization of shared bicycles, health-related mobile applications, and virtual reality tools within the realm of physical activity have assumed a growing significance in the daily routines of Chinese citizens amid the epidemic (13, 15). As we transition into the post-pandemic digital age, individuals can explore a variety of innovative technologies for enhancing their active mobility practices, such as utilizing smartphones for route planning, monitoring health metrics, and assessing traffic conditions to enhance the efficiency and safety of their travel experiences. At the organizational level, businesses and entities have the capacity to facilitate active mobility by implementing strategies such as flexible work hours and telecommuting policies. Moreover, organizations can enhance active transportation by offering amenities like bicycle parking facilities and financial assistance.

On a socio-political front, urban administrators should prioritize the development of bicycle lanes and pedestrian pathways to establish a secure and accessible environment for active transport. Additionally, drawing from past health crises, government bodies and academic institutions are encouraged to advocate for the advantages of active transportation through various channels such as media campaigns and community engagements to bolster public engagement and awareness. Through incentives like tax benefits, financial aid, and other supportive measures, governmental bodies can motivate individuals to adopt eco-friendly modes of travel. Lastly, it is imperative for policymakers and stakeholders to address the needs of marginalized demographics including the older adult, women, individuals with disabilities, and low-income populations by implementing structural reforms to mitigate disparities in the realm of active mobility.

The analysis of contemporary scholarly literature also provides valuable insights into potential directions for future research endeavors. Initially, the predominance of quantitative methodologies in existing studies suggests the potential for more comprehensive qualitative research to enhance our comprehension of various transformations and their enduring consequences within this domain. Subsequently, the prevalence of single-case studies underscores the need for further validation and enhancement of their findings through comparative analyses. Additionally, the examination of a broader range of social variables is warranted to address disparities in active travel during the post-epidemic period. Notably, the absence of studies focusing on the genuine “post-epidemic” era in China, commencing in late 2022, highlights a critical gap in the literature. The purported “post-epidemic” periods in current studies pertain to the recovery period, characterized by the persistence of certain anti-epidemic measures. Encouraging subsequent investigations that can shed light on emerging developments and transformations following the complete relaxation of restrictions will be instrumental in advancing our understanding of promoting active, healthy, and sustainable mobilities in the forthcoming era.

## Author contributions

SD: Conceptualization, Formal analysis, Methodology, Writing – original draft, Writing – review & editing. HT: Methodology, Writing – review & editing. HG: Visualization, Writing – review & editing.
